# Complete chloroplast genome of *Eurycorymbus Cavaleriei* (Sapindaceae), a tertiary relic rare tree

**DOI:** 10.1080/23802359.2019.1670113

**Published:** 2019-09-25

**Authors:** Zhang Chen, Oumeng Qiao, Bingbing Liu, Hailong Sun

**Affiliations:** a Institute of Loess Plateau, Shanxi University, Taiyuan, PR China;; b State Key Laboratory of Hydraulics and Mountain River Engineering, Sichuan University, Chengdu, PR China

**Keywords:** *Eurycorymbus cavaleriei*, chloroplast genome, phylogenetic analysis

## Abstract

The dioecious relic *Eurycorymbus cavaleriei* is a tertiary relict and only species of its genus within the family Sapindaceae. Using an Illumina platform, we sequenced its complete chloroplast (cp) genome. Our study reveals that *E. cavaleriei* has a typical cp genome of 158,537 bp in length, comprised of a large single-copy region (LSC) of 86,693 bp, a small single-copy region (SSC) of 18,012 bp, and two inverted repeat regions (IRs) of 26,916 bp, respectively. A total of 137 genes, 89 of which are protein-coding genes, 40 tRNA genes, 8 rRNA genes were identified. The overall GC content of the plastome is 38.0%. Phylogenetic analysis indicates that *E. cavaleriei* is closely related to the species of *Dodonaea viscosa*.

*Eurycorymbus cavaleriei* (Levl.) Rehd. et Hand. -Mazz., a rare tertiary relict and the only species of its genus within the family Sapindaceae, is endemic in the southern regions of China including Sichuan, Guangxi, Hunan, and Yunnan Provinces (Fu and Jin 1992). As a tertiary relic, it is an important species in the evolutionary study of the origin of flora in the tertiary period. However, because of the small (*n* < 200) and isolated populations, *E. cavaleriei* is treated as ‘vulnerable’ in China (Fu 1992) and it has been registered on the China Species Red List (Wang and Xie 2004). It is thus urgent to take effective measures to conserve this relic and rare species. Herein, we first report the complete chloroplast genome of *E. cavaleriei* based on Illumina paired-end sequencing data, which will aid in in-depth evolutionary study of the origin of flora in the tertiary period. The annotated cp genome of *E. cavaleriei* has been deposited into GenBank with the accession number MN421795.

In this study, *E. cavaleriei* was sampled from Chongqing of China, located at 109°20′49.0164″ E, 31°34′52.4208″ N. A voucher specimen (shl035) was deposited in the State Key Laboratory of Hydraulics and Mountain River Engineering, Sichuan University, Chengdu, China. The experiment procedure is as reported in Zhang et al. (2019). Around 2 Gb clean data were used for the cp genome de novo assembly by the program NOVOPlasty (Dierckxsens et al. 2017) and direct-viewing in Geneious R11 (Biomatters Ltd., Auckland, New Zealand). Annotation was performed with the program Plann (Huang and Cronk 2015) and Sequin (http://www.ncbi.nlm.nih.gov/).

The chloroplast genome of *E. cavaleriei* is a typical quadripartite structure with a length of 158,537 bp, which contained inverted repeats (IR) of 26,916 bp separated by a large single-copy (LSC) and a small single copy (SSC) of 86,693 bp and 18,012 bp, respectively. The cpDNA contains 137 genes, comprising 89 protein-coding genes, 40 tRNA genes, 8 rRNA genes. Among the annotated genes, 15 of them contain one intron (*atp*F, *ndh*A, *ndh*B, *rps*16, *rpoC*1, *pet*B, *pet*D, *rpl*16, *rpl*2, *trn*A-UGC, *trn*I-GAU, *trn*G-GCC, *trn*K-UUU, *trn*L-UAA, and *trn*V-UAC), and three genes (*rps*12 *clp*P and *ycf*3) contain two introns. The overall GC content of the plastome is 38.0%.

To identify the phylogenetic position of *E. cavaleriei*, phylogenetic analysis was conducted. A neighbor-joining (NJ) tree with 1000 bootstrap replicates was inferred using MEGA version 7 (Kumar et al. 2016) from alignments created by the MAFFT (Katoh and Standley 2013) using plastid genomes of 19 species. It showed the position of *E. cavaleriei* was closely related to the *Dodonaea viscosa* (Figure 1). Our findings will have valuable applications for chloroplast genetic engineering and evolutionary study of the origin of flora in the tertiary period.

**Figure 1.**
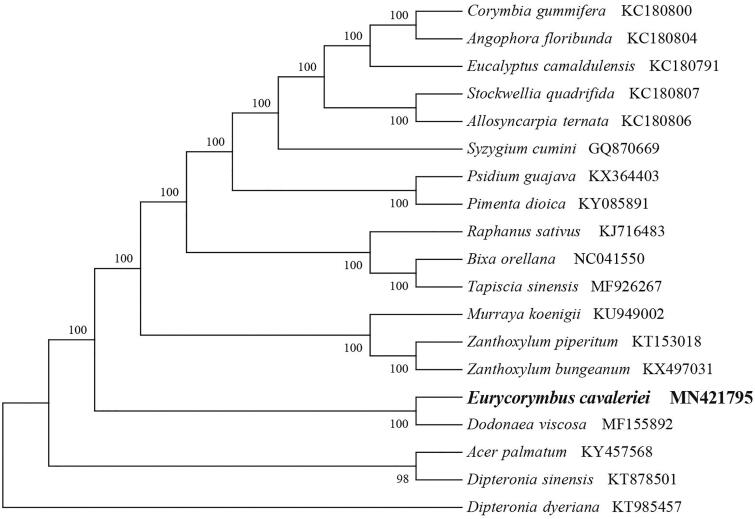
NJ phylogenetic tree of *E. cavaleriei* with 18 species was constructed by chloroplast plastome sequences. Numbers on the nodes are bootstrap values from 1000 replicates. *Dipteronia dyeriana* was selected as outgroups.
